# Editorial Commitment to Trust and Integrity in Science: Implications for Pain and Anesthesiology Research

**DOI:** 10.1213/ANE.0000000000007632

**Published:** 2025-09-23

**Authors:** Tonya M. Palermo, Didier Bouhassira, Karen D. Davis, Hugh C Hemmings, Robert W. Hurley, Joel Katz, Jaideep J. Pandit, Theodore J. Price, Michael E. Schatman, Stephan K. W. Schwarz, Dennis C. Turk, Marc Van de Velde, Matthew D. Wiles, Tony L. Yaksh, David Yarnitsky

**Affiliations:** From the 1 Department of Anesthesiology & Pain Medicine, University of Washington, Seattle, WA, USA; 2 Center for Child Health, Behavior & Development, Seattle Children’s Research Institute, Seattle, WA, USA; 3 Inserm U987, APHP, UVSQ, Paris-Saclay University, Ambroise Pare Hospital, Boulogne-Billancourt, France; 4 Department of Surgery and Institute of Medical Science, University of Toronto, Toronto, ON, Canada; 5 Krembil Brain Institute, University Health Network, Toronto, ON, Canada; 6 Department of Anesthesiology, Weill Cornell Medicine, New York, NY, USA; 7 Department of Anesthesiology, Translational Neuroscience (formerly Pharmacology), and Public Health Sciences, Pain Outcomes Lab, Wake Forest University School of Medicine, Winston-Salem, NC, USA; 8 Department of Psychology, York University, Toronto, ON, Canada; 9 Nuffield Department of Clinical Neuroscience, University of Oxford, Oxford, UK; 10 Nuffield Department of Anaesthesia, Oxford University Hospitals NHS Foundation Trust, Oxford, UK; 11 Department of Neuroscience and Center for Advanced Pain Studies, University of Texas at Dallas, Dallas, TX, USA; 12 Department of Anesthesiology, Perioperative Care, and Pain Medicine, NYU Grossman School of Medicine, New York, New York, USA; 13 Department of Population Health – Division of Medical Ethics, NYU Grossman School of Medicine, New York, New York, USA; 14 Department of Anesthesiology, Pharmacology & Therapeutics, The University of British Columbia, Vancouver, BC, Canada; 15 Department of Anesthesia, St. Paul’s Hospital/Providence Health Care, Vancouver, BC, Canada; 16 Department of Cardiovascular Sciences, Catholic University Leuven, Leuven, Belgium; 17 Department of Anesthesiology, University Hospitals Leuven, Leuven, Belgium; 18 Department of Academic Anaesthesia, Sheffield Teaching Hospitals NHS Foundation Trust, Sheffield, UK; 19 Centre for Applied Health and Social Care Research (CARe), Sheffield Hallam University, Sheffield, UK; 20 Department of Anesthesiology & Pharmacology, University of California, San Diego, San Diego, CA, USA; 21 Department of Neurology, Rambam Medical Center, and Laboratory of Clinical Neurophysiology, Technion Faculty of Medicine, Haifa, Israel.

## INTRODUCTION

We are a group of journal editors dedicated to advancing discoveries and innovations in basic, translational, and clinical research across anesthesiology and pain-related disciplines, which play a crucial role in reducing the burden of pain, improving health, enhancing perioperative outcomes, and optimizing healthcare delivery. Across scientific disciplines, concerns have been raised about research quality and trustworthiness.^1,2^ While these challenges are not unique to pain and anesthesiology research, we recognize this as a judicious opportunity to raise awareness and collaborate across our journals to align and strengthen initiatives to enhance research integrity, trust, and impact across our field.

In a 2005 landmark paper, Ioannidis concluded with the dramatic and troubling assertion that “most published research findings are false,” stimulating a large focus in the biomedical research community on understanding issues of integrity, reproducibility, and replication that continues to be relevant to this day.^3^ Indeed, there are many instances in which authors, institutions, funders, publishers and journals have failed to embody the core values that produce trustworthy science. The trustworthiness of research is affected by both intentional actions (e.g., fabrication and falsification of data, lack of rigor, image manipulation) and unintentional actions (e.g., inadequate oversight, awareness and understanding of both technical and scientific issues). Most concerning are instances of research misconduct including fabrication, falsification, or plagiarism sometimes revealed by failure to replicate or reproduce results, duplication of publications, a rise in the number of retractions,^4,5^ and calls for larger numbers of papers to be retracted (e.g.,^2^). In support of Ioannidis’s disquiet, some reviews (e.g.,^6,7^) report low replication rates of positive findings in the social and life sciences across clinical trials, epidemiological research, and molecular studies.

In anesthesiology specifically, low agreement has been found between randomized clinical trials (RCTs) and meta-analytic findings for clinical pain interventions, where positive findings in meta-analyses were often not confirmed by subsequent large RCTs. For example, using individual patient data from RCTs published in *Anaesthesia*, Carlisle^8^ demonstrated that almost half of the databases had false data as detected from the duplication of figures, tables, and other data from published work; the duplication of data in the rows and columns of spreadsheets; impossible values; and incorrect data analytic strategies and calculations.

Reproducibility, clinical validity, and utility in pain and anesthesiology research are often compromised by non-representative samples (e.g., limited representation on characteristics such as race, ethnicity, age, sex/gender, or socioeconomic status that do not match population-level data of those most affected by pain),^9–11^ reliance on surrogate outcomes with limited clinical relevance, underutilization of common data elements and core outcome sets, underpowered studies prone to false-negative results, and flawed statistical analysis plans that generate misleading conclusions.^12^

To ensure integrity of the literature, retraction of articles may be necessary due to such issues as major errors, data fabrication, plagiarism, or unethical research practices. Authors are encouraged to identify errors in their own work and may request a corrigendum to correct the literature. However, when ethical issues are brought to a journal’s attention, they have a duty to investigate, and when there is conclusive evidence, to impose a retraction to alert readers that the findings and conclusions cannot be relied upon.^13^ Retractions, when reported, can have a widespread impact due to the interconnectedness of studies attributed to the same authors.^14^ In the field of anesthesiology, the Retraction Watch Leaderboard^15^ indicates four of the top ten authors are anesthesiologists, and two of these individuals occupy the top two positions (https://retractionwatch.com/the-retraction-watch-leaderboard/. Systematic reviews have summarized characteristics of retracted publications for research misconduct in pain (e.g.,^16^) and anesthesiology research (e.g.,^17^). Concerns regarding retractions in all scientific fields are particularly noteworthy because they undermine trust in science, can have a lasting impact on conclusions made about treatments and, ultimately, impact clinical practice. In one study by O’Connell *et al*.,^18^ a set of 8 untrustworthy trials (i.e., identified due to concerns including data anomalies and implausible results), in spinal pain was determined to substantially impact the results of subsequent recommendations made in systematic reviews and international clinical practice guidelines in management of spinal pain.

Meta-research studies regarding open science practices highlight critical remaining gaps across many fields in reproducible research practices, open access data, and availability of protocols.^1^ In 2018, Lee *et al*.^19^ examined open science efforts in the pain field including preregistration of trials, sharing code, data, reproducible workflows, and the use of reporting guidelines. Among ten pain journals, a low level of engagement with open and transparent research policies was identified at that time. Cashin *et al*.^20^ also reviewed the policies of ten leading pain journals and determined that there were few journal policies adhering to transparency standards for review and publication. These observations have fueled many recent efforts and initiatives in open science including in pain and anesthesiology research.

Open and transparent research practices as embodied in the “open science” movement provide a more complete and accurate report of the research conducted and what was found, and share important aspects of the research process (e.g., availability of study materials, data and code).^21^ Trust and transparency are interwoven because when research is conducted and reported openly and transparently it increases confidence in the findings by enabling verification, replication, and critical appraisal.

For pain science to advance with groundbreaking discoveries and translation into clinical impact, it is important to produce high-quality, trustworthy research. Building on their earlier recommendations, O’Connell and colleagues^22^ recently presented a comprehensive framework for building trustworthy pain research called ENhancing TRUSTworthiness in Pain Evidence (ENTRUST-PE). The ENTRUST-PE framework conceptualizes the construct “Trustworthiness” of research to be supported by seven core values (**see Figure 1**^22^):

**Figure 1.**
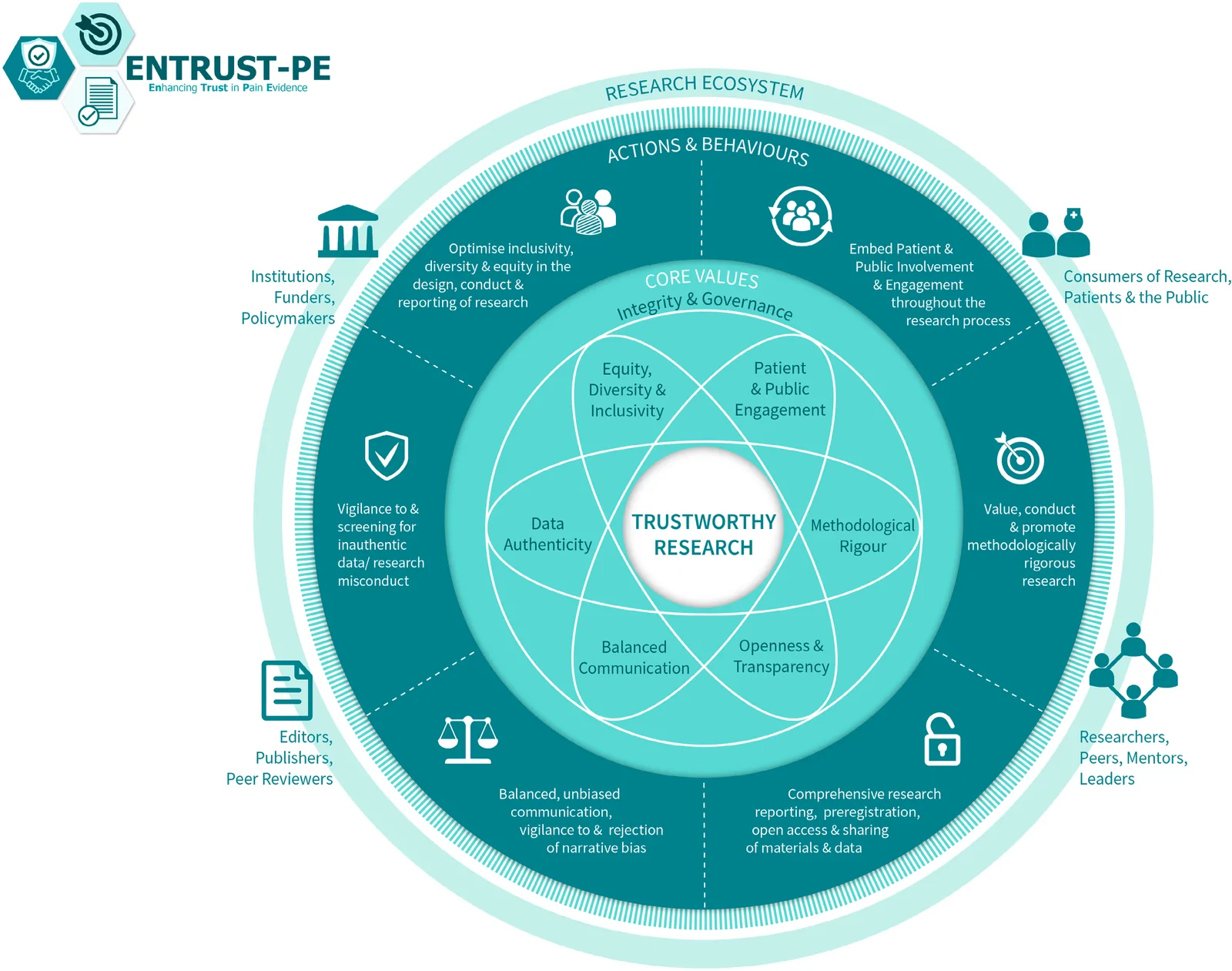
The ENhancing TRUSTworthiness in Pain Evidence framework (ENTRUST-PE). Reproduced with permission from O’Connell *et al.*^22^

1. Governance and Integrity (e.g., follow principles of research integrity and comply with regulatory guidelines, disclose conflicts of interest);
2. Equity, Diversity, and Inclusivity (e.g., plan strategies to maximize inclusivity at the preparation and initiation of the research);
3. Patient and Public Involvement and Engagement (e.g., embed partnership with people with lived experience throughout the research process);
4. Methodological Rigor (e.g., value, conduct, and promote high-quality methodologically rigorous research including in clinical studies with a focus on patient-centered outcomes, adequate power, and an appropriate analysis plan);
5. Transparency and Openness (e.g., adopt open research practices that include sharing of data, materials, and code);
6. Balanced Communication (e.g., report results accurately and comprehensively irrespective of the finding); and
7. Data Authenticity (e.g., commit to timely correction or removal of errors in the published literature).

Recommendations were made for short-term as well as more extended-term actions and behaviors for several different stakeholder groups (e.g., researchers, institutions, publishers, funders, policymakers and regulators, peer reviewers) to support trustworthy research within each of the core values of ENTRUST. These recommendations are intended to guide the development of a strategy for enhancing trustworthy research, rather than serving as a mandated policy.

From the perspective of engagement with our journals, here we focus on recommendations for researchers and editors/publishers.

## GUIDANCE FOR RESEARCHERS WHO PRODUCE, REVIEW, AND CONSUME RESEARCH

We strongly recommend that researchers thoroughly review the proposed framework, which we as editors endorse, and explore the full suite of resources available through the ENTRUST-PE network project. These can be accessed at https://entrust-pe.org and on the Open Science Framework (https://osf.io/cua7g/?view_only=ec1d9e6b1d774dbca9306ff5ae4dec67). The initiative is designed to support researchers to understand how to conduct and report science in a manner that enhances the transparency and trustworthiness of their work. By following these recommendations, researchers can provide the highest quality of research and facilitate confidence in pain science. Moreover, peer reviewers and consumers of research can be alert to potential issues of methodological rigor, transparency, lack of equity and inclusivity, and markers of potential data inauthenticity or research misconduct that play a critical role in raising concerns to editors and publishers when these are identified. Recently, both the International Association for the Study of Pain (IASP)^23^ and the European Pain Federation (EFIC)^24^ endorsed the ENTRUST-PE initiative, recognizing that trustworthy research not only benefits investigators and healthcare professionals but also serves patients and the public by promoting science that produces more effective pain management strategies.

**Table 1.** What Change Can I Make Now? Reproduced with permission from O’Connell et al.^22^

**Research Integrity and Governance**	Act consistently in alignment with the principles and values of research integrity. Be aware of local and wider research integrity and governance policies and act in alignment with those. Senior investigators: lead by example.
**Equity and Inclusivity**	When reporting research: • comprehensively report sample characteristics • adopt inclusive language, • use accurate interpretations of constructs of race, ethnicity, sex and gender • clearly make and report efforts to promote diversity and inclusion of study samples
**Patient and Public Involvement and Engagement**	Engage diverse potential patient and public partners before the project begins and involve them throughout the process. Plan PPIE at the very start (conception and planning) of the research process. Clearly report PPIE.
**Methodological Rigour**	Ensure the aims and questions of research are clearly conceptualised and communicated. Choose appropriate research designs for the research question. Provide adequate detail to reproduce study methodology.
**Transparency and Openness**	Pre-register your research, regardless of design. Update registrations with modifications to plans and results.
**Balanced Communication**	Report all planned results regardless of the findings. Consider the range of possible alternative interpretations as well as study limitations in your interpretation of study findings.
**Data Authenticity**	Draw attention to any errors in your work and issue corrections in a full, transparent and timely fashion.

For a concise, actionable summary, we reproduce the guidance provided by O’Connell *et al*.,^22^ which outlines practical suggestions researchers can implement immediately to align with the core values of the ENTRUST-PE framework (see **Table 1**).

## JOURNAL INITIATIVES

As editors of journals in the fields of pain science and anesthesiology, we wish to amplify the ENTRUST-PE framework^22^ and support efforts to promote, teach, and enforce principles and values underpinning high quality and trustworthy research. Here, we highlight four areas where we collectively aspire to take a leadership role in enhancing the trustworthiness of research in the journals we serve.

1. **Evaluate journal policies on transparency and openness to inform potential improvements.**

As highlighted in several prior reviews,^19,20^ journals can use existing tools to conduct self-assessments of their policies and procedures. Tools have been developed to facilitate transparency including the Transparency and Openness Evaluation Tool^20^ and the Centre for Open Science (COS) Transparency Factor.^25^ As a first step, pain and anesthesiology journals can sign on to COS as signatories (if they are not already) to express support of transparency and openness principles. In addition, the Transparency and Openness Factor metric provides information on where opportunities exist for improvement, which can contribute to decision making and policy development by Editors and Publishers to improve transparency and openness. For example, this can guide changes to journals^26^ along such areas as research pre-registration where appropriate, reporting guidelines, open data analytic codes and materials, transparent reporting of authorship contributions, and defining the role of the corresponding author as the point of contact for accountability and transparency.

We plan to undertake an updated and coordinated self-assessment process across our 15 journals using the procedures outlined by Cashin *et al*.^20^ This will provide a critical update on current engagement efforts with transparency standards across a larger number of pain and anesthesiology journals. Such an assessment will provide the journals with a list of potential areas for improvement to guide their efforts.

(2) **Gain access to automated tools to improve transparency and trustworthiness, while fostering innovation in new methodologies.**

Innovations are needed to support a range of automated processes to enhance transparency and integrity. At present, multiple checks of transparency and trustworthiness are conducted manually by reviewers and editorial teams. Journals can carry out protocols in the work flow *prior* to the initiation of peer review around many indicators for quality, trustworthiness, and ethics concerns such as possible image manipulation, internal inconsistencies in referral to figures and tables, text plagiarism, adherence to reporting checklists, registration of systematic reviews, identifying discrepancies between research registrations (e.g., ClinicalTrials.gov) and reporting of clinical trial outcomes, and the inclusion of relevant animal and human review board approvals, to name a few. One example of checking for random sampling in RCTs is the method suggested by Carlisle and colleagues, but (a) this is labor-intensive and (b) does not apply where recruitment has not been entirely random.^27,28^ Although there are automated processes to check for duplicate text, there are none yet to assist with these data integrity checks, and this requires dedicated staff effort. In this regard, several publishers/journals have introduced advanced technology (i.e., artificial intelligence) to detect duplicate manuscript submissions across all their respective journal platforms. Others have initiated “flag alerts” for authorships that include individuals who have been associated with multiple manuscript retractions. Additional automated processes are needed to help authors, reviewers, and editors to standardize more thorough yet efficient approaches to enhance transparency of reporting and enhance trustworthiness of published work.

Several approaches can be used to identify areas for improvement in this area. For example, we can engage in robust discussions with our publishers to emphasize the importance of automated tools, checks, and alerts, and advocate for their implementation in our journals. In addition, we can continue to advocate for adequate staffing to enable the critical checks needed for pre-review of submissions by the journal, which requires explicit formal training of a stable journal staff. While using advanced technology and providing journal staff entails a heightened responsibility of the publisher with possible financial consequences, it increases our confidence in the integrity of the research and builds trust in our science. We can also provide guidance and when possible, share resources (e.g., “how to guidance”) with our authors to enhance their own knowledge of tools to increase trustworthy science. For example, some reference management software (e.g., Zotero [Corporation for Digital Scholarship, Vienna, VA, USA], EndNote™ [Clarivate, London, UK]) have capabilities to check references for retractions.^29^

(3) **Create a platform for collaboration among editors of leading pain and anesthesiology journals**

This editorial highlights a significant collaboration among editors of leading pain and anesthesiology journals, which can serve as a foundation for continued engagement. We suggest holding online annual meetings and developing other platforms for information exchange for this group to discuss emerging trends, ethical concerns, and resource sharing. This may also serve as a forum for discussing general or specific integrity concerns and addressing the removal of inauthentic data from the literature, while ensuring confidentiality and privacy are upheld. We also recognize that there are barriers to engaging in transparency and integrity standards and anticipate initiating dialogue to better understand these barriers and how journals can support authors without increasing burden.

(4) **Offer educational opportunities and resources to professional societies, forums, journal reviewers, and early-career professionals**

Journals can be an important resource to guide and teach researchers and consumers about transparency and integrity standards, and we see several opportunities to make an impact. For example, one opportunity to introduce standards for trustworthiness is through the system adopted by several of our journals for manuscript review mentorship and editorial fellowship that provides tutorials, training, and experience reviewing or managing manuscripts. Moreover, we can leverage our partnerships with the professional societies that are associated with many of our journals to offer training and instruction on transparency and integrity. This could include professional development programs for reviewers, as well as early-career faculty (e.g., North American Pain School), and offerings developed by groups such as the International Association for the Study of Pain’s Early Career Network, (https://www.iasp-pain.org/early-career-network/), and by setting expectations for presenting and sharing research at scientific meetings (e.g., checking for retractions of any published studies discussed in presentations). Our journals can help disseminate information on tools targeting researchers directly^30^ that can be made available to authors in a toolkit to assist them in pursuing values of openness and integrity. For example, statistical assessment tools to assess the accuracy of reported findings may be implemented by running simple, automated error checks, such as using the StatCheck tool.^31^ It should be stressed that increasing the education provided enhances quality, reliability, and integrity.

## CONCLUSIONS

Ultimately, as a community of scientists and clinicians in pain and anesthesiology, we must recognize that trust is a dynamic and multifaceted concept. It requires ongoing effort to maintain, once lost is hard to regain, and it is built through consistent actions and open communication. Resources are available through the ENTRUST-PE framework that can guide actions and values to promote trust and integrity. These principles apply to all scientific fields beyond those that are pain-related and we encourage other specialties to harmonize such efforts. As editors, we will work together to advance the trustworthiness of research through upholding rigorous standards, ethical conduct, and open dialogue. By doing so, we can strengthen the foundation of trust in research and ensure that anesthesia and pain science continue to optimally inform care for people undergoing anesthesia or living with pain.

## DISCLOSURES

None of the authors have disclosures directly related to this work. TMP receives research grant funding from NIH, provides consultation to TriveniBio, and serves as Editor-in-Chief, *The Journal of Pain*. DB serves as Editor-in-Chief, *European Journal of Pain.* KDD serves as Editor-in-Chief, *PAIN.* HCH receives research grant funding from NIH and serves as Editor-in-Chief, *British Journal of Anaesthesia.* RWH has received cooperative research grants from NIH and Nevro, Inc., provides expert/consulting to State Farm, and serves as Editor-in-Chief, *Pain Medicine*. JK is the Research Director of ManagingLife and serves as Editor-in-Chief, *Canadian Journal of Pain*. JJP is Editor-in-Chief, *Anesthesia & Analgesia* (and was not involved in the handling of this manuscript). TJP is a co-founder of and holds equity in 4E Therapeutics, NuvoNuro, PARMedics, Nerveli, and Doloromics, has received research grants from AbbVie, Eli Lilly, Grunenthal, Evommune, GSK,l\ Hoba Therapeutics, and The National Institutes of Health, and serves as Editor-in-Chief, *Neurobiology of Pain.* MVdV has received honoraria for lectures and/or consultancy from CSL Behring, CSL Vifor, BBraun, Werfen, Viatris, CAF-DCF and Aquettant, and serves as Editor-in-Chief, *European Journal of Anesthesiology.* MS is Senior Medical Advisor, APURANO Pharma, and serves as Editor-in-Chief, *Journal of Pain Research.* SKWS holds the Dr. Jean Templeton Hugill Chair in Anesthesia, supported by the Dr. Jean Templeton Hugill Endowment for Anesthesia Memorial Fund (Faculty of Medicine, The University of British Columbia, Vancouver, BC, Canada) and gratefully acknowledges the Department of Anesthesia, St. Paul’s Hospital/Providence Health Care (Vancouver, BC, Canada) for ongoing support. SKWS serves as Editor-in-Chief, *Canadian Journal of Anesthesia.* DCT holds the John and Emma Bonica Endowed Chair in Anesthesiology & Pain Research at the University of Washington, has received research grants from NIH and NIOSH, and has provided consultation to Vertex. DCT is also Associate Director of Analgesic, Anesthetic, and Addiction Clinical Trials Translations, Innovations, Opportunities, and Networks & Pediatric Anesthesia Safety Initiative (ACTTION/PASI). DCT serves as Editor-in-Chief, *Clinical Journal of Pain*. MDW serves as Editor-in-Chief, *Anaesthesia*. TLY is co-founder of Raft Pharmaceuticals, serves on the Scientific Advisory Board for Navega Therapeutics, and serves as the Editor-in-Chief for *Frontiers in Pain Research*. DY is a partner in a current Horizons grant, holds equity in BrainsGate and Theranica, and receives honorariums from Dr Reddy and from IASP. DY serves as Editor-in-Chief, *PAIN Reports.*
